# Spatial Ventilation Inhomogeneity Determined by Electrical Impedance Tomography in Patients With Chronic Obstructive Lung Disease

**DOI:** 10.3389/fphys.2021.762791

**Published:** 2021-12-13

**Authors:** Inéz Frerichs, Livia Lasarow, Claas Strodthoff, Barbara Vogt, Zhanqi Zhao, Norbert Weiler

**Affiliations:** ^1^Department of Anesthesiology and Intensive Care Medicine, University Medical Center Schleswig-Holstein, Kiel, Germany; ^2^Department of Biomedical Engineering, Fourth Military Medical University, Xi’an, China; ^3^Institute of Technical Medicine, Furtwangen University, Villingen-Schwenningen, Germany

**Keywords:** EIT, lung imaging, obstructive lung disease, airway obstruction, forced ventilation maneuver, pulmonary function testing, functional imaging, electrical bioimpedance

## Abstract

The aim of this study was to examine whether electrical impedance tomography (EIT) could determine the presence of ventilation inhomogeneity in patients with chronic obstructive lung disease (COPD) from measurements carried out not only during conventional forced full expiration maneuvers but also from forced inspiration maneuvers and quiet tidal breathing and whether the inhomogeneity levels were comparable among the phases and higher than in healthy subjects. EIT data were acquired in 52 patients with exacerbated COPD (11 women, 41 men, 68 ± 11 years) and 14 healthy subjects (6 women, 8 men, 38 ± 8 years). Regional lung function parameters of forced vital capacity (FVC), forced expiratory volume in 1 s (FEV_1_), forced inspiratory vital capacity (FIVC), forced inspiratory volume in 1 s (FIV_1_), and tidal volume (V_*T*_) were determined in 912 image pixels. The spatial inhomogeneity of the pixel parameters was characterized by the coefficients of variation (CV) and the global inhomogeneity (GI) index. CV and GI values of pixel FVC, FEV_1_, FIVC, FIV_1_, and V_T_ were significantly higher in patients than in healthy subjects (*p* ≤ 0.0001). The ventilation distribution was affected by the analyzed lung function parameter in patients (CV: *p* = 0.0024, GI: *p* = 0.006) but not in healthy subjects. Receiver operating characteristic curves showed that CV and GI discriminated patients from healthy subjects with an area under the curve (AUC) of 0.835 and 0.852 (FVC), 0.845 and 0.867 (FEV_1_), 0.903 and 0.903 (FIVC), 0.891 and 0.882 (FIV_1_), and 0.821 and 0.843 (V_T_), respectively. These findings confirm the ability of EIT to identify increased ventilation inhomogeneity in patients with COPD.

## Introduction

Chronic obstructive pulmonary disease (COPD) is a highly prevalent lung disease associated with high mortality (GBD 2019 Diseases and Injuries Collaborators, 2020). COPD is characterized by the presence of chronic and mostly irreversible air flow limitation which is caused by two mechanisms: (1) increased airway resistance due to airway narrowing and (2) reduced elastic recoil of the lung tissue in the presence of emphysema (Moreno et al., 1986). The disease is progressive and the patients can be allocated to different COPD severity stages using the GOLD and the ABCD classification schemes (Vogelmeier et al., 2017). Although these classifications are established, they do not always allow reliable predictions of health-related outcomes and mortality (Soriano et al., 2015).

Pulmonary function testing using spirometry is the standard method for assessing the lung status of patients suffering from COPD. The spirometric examinations are standardized regarding the data acquisition, quality criteria, analyses, and reporting (Miller et al., 2005; Graham et al., 2019). The forced full expiration is an established ventilatory maneuver that the subjects are asked to perform during the spirometric examination. This maneuver carried out after preceding deep inspiration to total lung capacity, renders the well-known lung function parameters of forced expiratory volume in 1 s (FEV_1_), forced vital capacity (FVC), and their ratio FEV_1_/FVC. These parameters are used to stage the disease severity, to follow the disease progression and the effects of therapy in patients with COPD. Reference values based on worldwide examinations of tens of thousands of healthy subjects of different genders, ages, and ethnicities are available (Quanjer et al., 2012a,b).

The full medical assessment of patients with COPD also requires the use of medical imaging techniques. Conventional radiological methods of chest radiography and computed tomography are preferentially used to visualize the morphological structural changes in the lung tissues, especially regarding the diagnosis of emphysema (Miniati et al., 2008). These examinations are carried out discontinuously; often after the exacerbation of the disease, the patients need to visit the radiology department and they are exposed to radiation.

The question arises whether the patients with COPD would not benefit from additional diagnostic and monitoring methods allowing not only better phenotyping and improved staging of the patients but also earlier identification of disease exacerbations with timely adjustment of therapy and potential prevention of hospital admissions. Over the recent approximately 10 years, a few studies were carried out in patients with COPD (Vogt et al., 2012, 2016; Milne et al., 2019; Zhao et al., 2020a; Lasarow et al., 2021) and patients with other chronic lung diseases (Zhao et al., 2012, 2013; Frerichs et al., 2016; Krueger-Ziolek et al., 2016; Lehmann et al., 2016), demonstrating that the method of electrical impedance tomography (EIT) might offer a potentially interesting option for the assessment of regional pulmonary function combined with imaging.

The number of clinical studies studying the performance of EIT in identifying airway obstruction in patients suffering from COPD is still rather limited. In contrast to the relatively broad interest in EIT applications in mechanically ventilated patients (Kobylianskii et al., 2016; Frerichs et al., 2017; Zhao et al., 2020b; Bronco et al., 2021), there exists only a handful of research groups investigating the use of EIT in spontaneously breathing patients with COPD. Hence, the general data availability from this patient group is low and many aspects of data acquisition, analysis, and interpretation are not clarified yet. In view of the scarce research results in this field, we have initiated a clinical study with the intention to examine whether the presence of ventilation inhomogeneity in patients suffering from COPD and hospitalized due to disease exacerbation could be obtained by EIT not only from the conventional forced full expiration maneuvers but also from the preceding forced inspiration maneuvers and quiet tidal breathing. In addition, we wanted to analyze whether the degrees of ventilation inhomogeneity calculated from these distinct ventilation phases were comparable among each other and whether the determined ventilation inhomogeneity was significantly higher compared with healthy subjects.

## Methods

### Study Participants

The study was conducted on 52 hospitalized patients suffering from exacerbated COPD (11 women and 41 men) and aged 68 ± 11 years (mean ± SD) with a body weight of 74 ± 17 kg and height of 174 ± 7 cm. A total of 17 patients were classified as GOLD IV stage, 21 as GOLD III, 13 as GOLD II, and 1 as GOLD I. Forty-eight patients were either past or current smokers. Twenty-three patients required intermittent oxygen therapy. Short- and long-acting muscarinic antagonists were documented in 28 and 31 patients, respectively, and short- and long-acting beta_2_-adrenergic agonists in 39 and 41 patients, respectively. A group of 14 healthy subjects (6 women, 8 men) with no history of lung disease, aged 38 ± 8 years (mean ± SD) with a body weight of 75 ± 15 kg and height of 176 ± 11 cm served as a reference group. The study was approved by the Ethics Committee of the Medical Faculty of the Christian Albrechts University in Kiel, Germany (D 444/16). All subjects gave their written informed consent to participate in the study.

All study participants underwent conventional pulmonary function testing using spirometry (MasterScreen, CareFusion, Höchberg, Germany). Afterward, the subjects were examined by EIT.

### Electrical Impedance Tomography Examination and Data Analysis

Sixteen self-adhesive electrodes (BR-50-L, Ambu, Ballerup, Denmark) were placed equidistantly around the chest in one transverse plane at the level of the 5–6th intercostal space at the parasternal line and one additional reference electrode on the abdomen. Raw EIT data were acquired with the Goe-MF II device (CareFusion, Höchberg, Germany) at a scan rate of 33 images/s using excitation currents of 5 mA_rms_ applied through adjacent electrodes. Image reconstruction was accomplished by the GREIT algorithm (Adler et al., 2009). The baseline for image reconstruction was obtained individually for each subject during quiet tidal breathing.

Electrical impedance tomography examinations were performed in seated subjects who were instructed not to speak and not to move their upper torso during the data acquisition. The subjects had their hands placed on their thighs. Data were recorded continuously during a period of quiet tidal breathing followed by a slow full expiration to residual volume with a subsequent forced full inspiration to total lung capacity and forced full expiration back to residual volume. Then, the subjects resumed tidal breathing and the recording was stopped.

**FIGURE 1:**
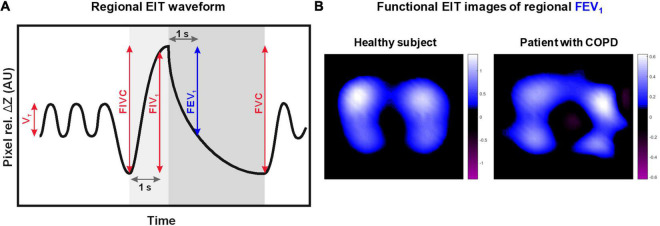
Schematic drawing of the electrical impedance tomography (EIT) waveform in one image pixel. The waveform reflects the regional lung volume changes over time during pulmonary function testing **(A)**. Five lung function measures analyzed from the periods of quiet tidal breathing, forced full inspiration, and forced full expiration are highlighted: tidal volume (V_T_), forced inspiratory vital capacity (FIVC), forced inspiratory volume in 1 s (FIV_1_), forced expiratory volume in 1 s (FEV_1_), and forced vital capacity (FVC). These measures are calculated in all image pixels and can be visually presented in form of two-dimensional maps called functional EIT images. Example functional EIT images **(B)** showing the distribution of regional FEV_1_ acquired in a healthy subject and a patient with chronic obstructive lung disease (COPD) are provided.

The EIT recordings were used to calculate one characteristic volumetric lung function parameter from the tidal breathing period, two parameters from the forced inspiration, and another two from the forced expiration per each image pixel (Figure 1). These parameters correspond to the same parameters that are obtained during conventional spirometry with the only difference being that their calculation is performed not only on just one waveform but on 912 EIT waveforms. The EIT waveform analysis was carried out offline as follows: a stable quiet tidal breathing period in the length of about 60 s was selected at first. Then, the consecutive inspiratory maxima and the expiratory minima of relative impedance changes (rel. ΔZ) in arbitrary units were identified in the waveform. The average differences between the rel. ΔZ values at these time points, corresponding to average tidal impedance variations and representing regional tidal volumes (V_T_), were calculated in all image pixels. Afterward, the differences between the maximum rel. ΔZ values achieved after the forced full inspiration to total lung capacity and the lowest rel. ΔZ values during the preceding maximum expiration to residual lung volume were calculated in all pixels. These values reflected the pixel values of forced inspiratory vital capacity (FIVC). Another parameter determined from the forced full inspiration was the forced inspiratory volume in 1 s (FIV_1_), calculated as the difference between the rel. ΔZ values after 1 s of forced full inspiration and the value at residual lung volume at the beginning of the inspiration limb of the maneuver in every image pixel. The forced full expiration rendered two parameters, FEV_1_ and FVC, which were calculated as the differences between the pixel rel. ΔZ values at total lung capacity directly before the onset of full forced expiration and the rel. ΔZ values after 1 s of expiration and the lowest rel. ΔZ values at the end of forced full expiration, respectively. Similar to the three already mentioned parameters, also FEV_1_ and FVC were obtained from all image pixels.

The calculated pixel values of all five parameters can be visually presented as two-dimensional maps with color-coding reflecting the magnitude of the pixel parameter values. Such plots are called functional EIT images in EIT terminology (Frerichs et al., 2017). For example, functional EIT images showing the distribution of pixel FEV_1_ values in a healthy subject and a patient with COPD are presented in Figure 1B.

To quantitatively characterize the dispersion of pixel values of FVC, FEV_1_, FIVC, FIV_1_, and V_T_ in the functional images, i.e., the heterogeneity of their spatial distribution, the respective coefficients of variation (CV), and the global inhomogeneity (GI) indices were determined in each patient and healthy subject. CV was calculated as the ratio of the SD to the mean pixel value of each parameter. The GI index was defined as the sum of all absolute differences between each pixel value and the median divided by the sum of all pixel values (Zhao et al., 2009). Finally, receiver operating characteristic (ROC) analysis was applied to assess the power of CV and the GI index of all five regional EIT lung function parameters to discriminate between the patients and the healthy subjects based on the calculation of the areas under the ROC curves (AUC).

### Statistical Analysis

Statistical analysis of the data was performed with GraphPad Prism 9.02 (GraphPad Software Inc., San Diego, CA, United States). Data are reported as mean values ± SD or median values with interquartile ranges. D’Agostino and Pearson’s test was used to assess the presence of normal distribution. Mann–Whitney *U* test was applied for comparisons between the groups of patients and healthy subjects. Friedman test (with Dunn’s test for multiple comparisons) and repeated measures ANOVA (with Bonferroni’s test for multiple comparisons) were used to assess the effect of the analyzed EIT parameter (i.e., FVC, FEV_1_, FIVC, FIV_1_, and V_T_) on the determined ventilation heterogeneity in patients and healthy subjects, respectively. Two-sided *p-*values smaller than 0.05 were considered significant. In the case of multiple comparisons, each *p-*value was adjusted as appropriate.

## Results

The results of conventional spirometry examinations are summarized in Table 1. They confirmed the severely deteriorated lung function in patients compared with healthy subjects, whose lung function was in the normal range. The spirometry values in subgroups of patients according to the documented GOLD severity stage are provided in Supplementary Table 1. They fell significantly with the disease severity (*p* < 0.0001). The respiratory rates during spontaneous tidal breathing directly preceding these examinations are given in Table 1, and they showed no significant differences between patients and healthy subjects.

**TABLE 1:** Results of conventional spirometry in the studied patients with chronic obstructive lung disease (COPD) and healthy human subjects.

	FEV_1_/FVC (*Z* score)	FEV_1_ (*Z* score)	FVC (*Z* score)	f_resp_ (breaths/min)
Patients	−3.09 ± 1.15^a^	−3.48 ± 1.07^a^	−2.57 ± 1.19^a^	18.0 ± 5.0
Healthy subjects	−0.38 ± 1.04	−0.22 ± 0.89	0.06 ± 1.27	17.1 ± 3.7

*Data are presented as mean ± SD.*

*FEV_1_, forced expiratory volume in 1 s; FVC, forced vital capacity; f_resp_, respiratory rate during quiet tidal breathing preceding the ventilation maneuver.*

*^a^p < 0.0001 vs. healthy subjects.*

**FIGURE 2:**
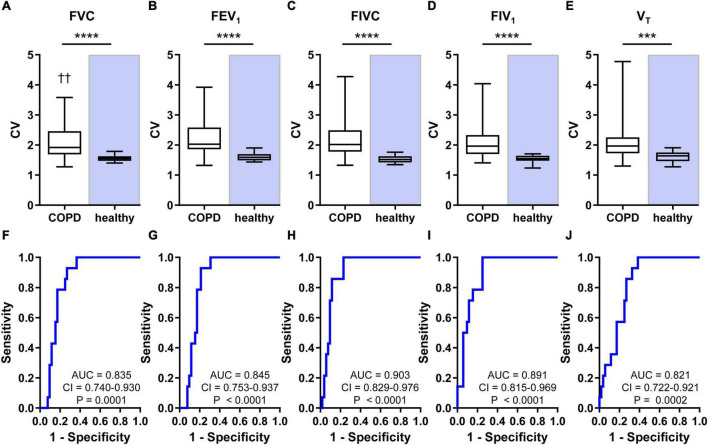
Heterogeneity of regional lung ventilation in patients with COPD and healthy human subjects and the results of receiver-operating characteristics (ROC) analyses. Coefficients of variation (CV) of the pixel values of FVC **(A)**, FEV_1_ **(B)**, FIVC **(C)**, FIV_1_ **(D)**, and V_T_ **(E)** are shown at the top. The ROC curves revealing the power of CV of the pixel values of FVC **(F)**, FEV_1_ **(G)**, FIVC **(H)**, FIV_1_ **(I)**, and V_T_ **(J)** to discriminate between the patients suffering from COPD and the healthy subjects are displayed at the bottom. Box and whisker plots show the minimum, 25% percentile, median, 75% percentile, and maximum values. Significant differences between the two groups are indicated: ****p < 0.0001; ***p = 0.0001; ††*p* < 0.01 (vs. FEV_1_). AUC, confidence interval (CI), and *p*-values are given in the bottom part of each ROC curve diagram.

**FIGURE 3:**
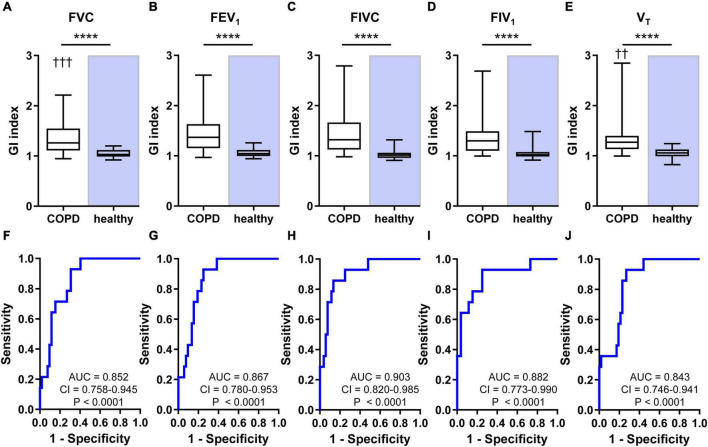
Heterogeneity of regional lung ventilation in patients with COPD and healthy human subjects and the results of ROC analyses. The values of GI indices calculated from the pixel values of FVC **(A)**, FEV_1_ **(B)**, FIVC **(C)**, FIV_1_ **(D)**, and V_T_ **(E)** are shown at the top. The ROC curves revealing the power of the GI indices calculated from the pixel values of FVC **(F)**, FEV_1_ **(G)**, FIVC **(H)**, FIV_1_ **(I)**, and V_T_ **(J)** to discriminate between the patients suffering from COPD and the healthy subjects are given at the bottom part of the figure. Box and whisker plots show the minimum, 25% percentile, median, 75% percentile, and maximum values. Significant differences between the two groups are indicated: ****p < 0.0001; ††*p* < 0.01 (vs. FEV_1_); † † †*p* < 0.001 (vs. FEV_1_). AUC, CI, and *p*-values are given in the bottom part of each ROC curve diagram.

The CV of pixel values of FVC, FEV_1_, FIVC, FIV_1_, and V_T_ exhibited higher values in patients than in healthy subjects (Figure 2). The differences were highly significant with *p* < 0.0001 for FVC, FEV_1_, FIVC, and FIV_1_ and *p* = 0.0001 for V_T_. The GI indices of pixel values of FVC, FEV_1_, FIVC, FIV_1_, and V_T_ were also higher in patients than in healthy subjects (all *p* < 0.0001) (Figure 3). The CV and GI values of all five regional EIT parameters were normally distributed in the healthy subjects but not in the patients with COPD.

The CV (*p* = 0.0024) and GI values (*p* = 0.0006) were affected by the analyzed ventilation parameter in patients but not in healthy subjects (*p* = 0.2425 and *p* = 0.6365, respectively). In patients, CV of regional FEV_1_ was significantly higher than the CV value of FVC, and GI of regional FEV_1_ was significantly higher than GI of FVC and V_T_. The multiple comparisons within the statistical analysis did not reveal any other significant differences between the CV and GI values of the five lung function parameters.

The ROC analysis showed that CV discriminated patients from healthy subjects with all AUC values higher than 0.821 (Figure 2) and GI with values higher than 0.843 (Figure 3). The AUC values of the CV of FVC, FEV_1_, FIVC, and FIV_1_ (all *p* < 0.0001) confirmed a slightly higher discriminative power of these EIT parameters compared with the CV value of V_T_ (*p* = 0.0002). The GI index was comparably discriminative for all five regional lung function parameters (all *p* < 0.0001).

## Discussion

Our study showed that the spatial distribution of ventilation determined by EIT was consistently more heterogeneous in patients with COPD than in healthy subjects. The increased heterogeneity of ventilation was detected during all analyzed breathing phases of quiet tidal breathing, forced full inspiration, and forced full expiration. The CV and GI values of all five calculated pixel lung function measures were highly significantly increased compared with the respective values in healthy adults. Overall, the results confirmed the previous observations demonstrating that EIT can determine the pathologically increased ventilation heterogeneity in chronic obstructive disease (Vogt et al., 2012; Zhang et al., 2018) and extended the former findings by showing that this information can be derived by EIT not only from the forced full expiration maneuver but also from forced full inspiration and even quiet tidal breathing.

The latter observation is particularly intriguing because it supports the possibility of deriving information on the deteriorated lung function from EIT recordings acquired during quiet breathing and not only from the traditional forced full expiration maneuver. It is known that the global pulmonary function measures derived from the forced full expiration maneuver, such as FEV_1_ and FVC, are effort-dependent (Krowka et al., 1987; Park, 1988). Hence, their values are influenced by the motivating instructions of the examiner and the compliance of the patient. Such maneuvers also cannot be accomplished in some patients who do not or cannot cooperate, as in small children. Conventional spirometry does not provide information on the distribution of ventilation in the chest, but if it is confirmed in the future that this type of information accessible by EIT is of relevance in clinical decision-making, then the option of obtaining it from the patients without the necessity of any ventilation maneuver needs to be examined. At present, this idea is speculative because the data supporting it are still scarce.

In the present study, the ROC curve generated from the CV and GI values of pixel V_T_ exhibited the smallest AUC among the five ROC curves, albeit it was still possible to differentiate the patients from the healthy subjects at a high significance level. The ROC analysis carried out in the already mentioned earlier study (Vogt et al., 2012) also showed a slightly smaller AUC for V_T_ than for FEV_1_ and FVC. Interestingly, the discriminative power of the currently determined CV of V_T_, FEV_1_, and FVC with AUC values of 0.82, 0.85, and 0.83, respectively, was higher than in the earlier study where the respective values were 0.71, 0.77, and 0.73 (Vogt et al., 2012). This finding can be explained by the differences between the study patient populations. In the current study, patients with COPD hospitalized mainly due to exacerbated disease were examined, whereas, in the former study, patients with stable COPD in an outpatient clinic were included. Hence, it may be presumed that the exacerbated disease affected the regional lung function negatively and further increased the ventilation inhomogeneity.

An issue that still needs to be investigated is how good EIT is in identifying ventilation heterogeneity in patients at an early stage of COPD with mild airway obstruction and if EIT data acquired during quiet breathing in these patients are still reliable in identifying the existing lung pathology. Most of the studies performed so far, including the current one, examined no or a minimum number of patients with low disease severity (Vogt et al., 2012, 2016; Milne et al., 2019). The only exception is the study by Zhang et al. (2018) in which 39% of the study population was represented by patients with early airway obstruction. That study was able to show that these patients could be discriminated from a healthy control group based on the most frequent pixel FEV_1_/FVC values. However, the patients were not examined during quiet breathing, and no quantitative analysis of the degree of ventilation heterogeneity based on an EIT measure characterizing the dispersion of the pixel lung function parameters (such as the CV and GI values used in the present study) was performed. Thus, the evidence supporting the use of EIT to identify ventilation heterogeneity during quiet breathing in patients with early COPD is generally lacking.

An interesting finding obtained in our study was that the spatial distribution of ventilation was not independent of the analyzed breathing period in patients, meaning that the detected ventilation heterogeneity exhibited a slight dependency on the analyzed regional lung function parameter with the distribution of pixel FEV_1_ being more heterogeneous. This contrasted the findings in healthy subjects, in whom the spatial distribution of ventilation was independent of the analyzed EIT lung function parameter. It can be postulated that the irreversible changes in lung architecture encountered in COPD, that favor regional airway collapse during the forced blow with resulting airflow limitation, led to a somewhat more heterogeneous air distribution pattern in the lungs.

It has been previously presented that the forced inspiratory maneuver and the lung function measures derived from it, such as FIV_1_, might better assess the subjective perception of the lung function status by the patients and be more adequate to assess the therapy effects than FEV_1_ (Taube et al., 2000). In that sense, the finding that EIT can reliably determine the presence of spatially inhomogeneous ventilation distribution during forced inspiration is potentially clinically relevant. If FIV_1_ is more closely linked with the patients’ disease perception and dyspnea (Taube et al., 2000), then also the EIT measures of ventilation distribution based on the forced inspiration maneuver might be more suitable in patient phenotyping and the assessment of treatment than the measures based on forced full expiration. As already mentioned above, also this potential approach of using EIT needs to be examined in further studies and validated.

Finally, we would like to address a few limitations of our study. (1) The study had an exploratory and hypothesis generating design. This is understandable in view of the still rudimentary research on the use of EIT for the characterization of regional lung function in COPD. Nonetheless, several of the potentially interesting study findings will need to be confirmed and validated in future studies. (2) The healthy subjects within the control group in our study were not age-matched with the group of patients. This might have caused a bias toward more homogeneous ventilation distribution in the younger healthy subjects. However, a previous study has demonstrated only minimum differences between lung-healthy young and elderly adults regarding the spatial distribution of ventilation (Vogt et al., 2012). The young and elderly in that study were of comparable age to our study groups, and CV was also used as the EIT measure of ventilation heterogeneity. (3) A reference group of patients without a chronic lung disease but suffering from acute lung disease was not included. (4) Our study cohort did not allow us to analyze the relationship between the GOLD stage and EIT measures because none of the hospitalized patients was in a stable COPD phase and the detected ventilation heterogeneity, therefore, cannot be attributed solely to the underlying COPD disease stage. Additionally, it reflected the effects of acute exacerbation on ventilation distribution. Our study design did not allow us to differentiate between these two origins of increased ventilation heterogeneity. The patient cohort was also relatively small and the less severe stages were underrepresented. (5) Our study was not suitable for analyzing gravity-dependent phenomena in the distribution of regional lung mechanics and ventilation. All subjects were examined in the upright posture, and all measurements were conducted with the electrodes placed in just one transverse plane. This means that the studied lung section was located in the same plane with respect to the gravity vector. However, the gravity-dependent phenomena can be assessed by EIT when the studied subjects are in a horizontal posture, as demonstrated in healthy subjects (Frerichs et al., 1996), experimental animals (Kunst et al., 2000), or patients with respiratory failure (Victorino et al., 2004; Pulletz et al., 2012; Scaramuzzo et al., 2020). Alternatively, the subjects can be examined with the electrodes placed sequentially at first in the cranial and then the caudal chest location (or vice versa) (Reifferscheid et al., 2011). No EIT devices approved for clinical use allow simultaneous examinations in multiple chest planes at the moment, although a few experimental data became available since the early pioneering study (Metherall et al., 1996). (6) A relatively old EIT hardware was used during the examinations of the study participants. This EIT device offered the maximum scan rate of 33 images/s which is lower compared with the modern devices that achieve scan rates of typically around 50 images/s. The available scan rate was still sufficient for the analysis of the intended EIT parameters but we cannot completely exclude that it presented a certain drawback, especially in the case of the analysis of regional FEV_1_. This measure depends on the exact identification of the onset of the forced expiration. Since the air flow rates are very high, with peak rates reaching 10–12 l/s in the healthy, even very small imprecisions in identifying the correct time points may impact the calculated FEV_1_ values. We do not consider this potential effect to have substantially affected the current results, since the used data analysis software allowed the manual adjustment of the automatically identified time points in case of need. (7) Single self-adhesive electrodes were used in our study instead of compact electrode belts applied in the modern EIT devices. The placement of individual electrodes on the chest is more time-consuming than the use of electrode belts but it posed no major drawback in view of the study design. Compared with the belts, which have been shown to influence the volumetric measures of lung function, for example, global FVC, determined by spirometry in patients to some extent (Zhang et al., 2020), the single electrodes might even have been advantageous because they do not limit the movements of the rib cage. On the other hand, the skin contact of self-adhesive electrodes may be insufficient, especially in male subjects with chest hair, impeding the quality of EIT data recordings. Thus, solutions for electrode interfaces specifically designed for EIT examination of patients who are spontaneously breathing in upright body position and performing deep ventilation maneuvers are needed. First attempts at creating such electrode interfaces that are integrated into wearable vests have been proposed (Rapin et al., 2015, 2019; Frerichs et al., 2020), which might mature in the future and allow even remote use of EIT for home monitoring of patients with COPD. 8) Compared with the widespread use of spirometry, the use of EIT for the assessment of lung function deterioration is still at a very early stage of development.

## Conclusion

The spatial distribution of regional volumetric lung function measures determined by EIT is heterogeneous in patients suffering from COPD and consistently higher than in healthy subjects. EIT is able to reliably discriminate the patients with exacerbated COPD from healthy subjects using the CV and GI values of pixel V_T_, FIV_1_, and FIVC obtained from EIT examinations carried out during quiet tidal breathing and forced full inspiration and not only of pixel FEV_1_ and FVC calculated from ET images recorded during the conventional forced full expiration maneuvers. These findings support further research on the use of EIT in spontaneously breathing patients with COPD, nonetheless, the role of EIT in the diagnostics, therapy monitoring, prognostics, and clinical outcome assessment of patients with COPD remains yet to be established. With COPD presenting a major public health problem, this recently identified research field, exploring the regional lung function testing with EIT in patients with this disease, seems reasonable.

## Data Availability Statement

The raw data supporting the conclusions of this article will be made available by the authors, without undue reservation.

## Ethics Statement

The studies involving human participants were reviewed and approved by the Ethics Committee of the Christian Albrechts University in Kiel, Kiel, Germany. The patients/participants provided their written informed consent to participate in this study.

## Author Contributions

IF, BV, and NW designed the study. LL, CS, and BV conducted the study. IF, LL, and ZZ analyzed the study. IF wrote the first draft of the manuscript. All authors reviewed the manuscript, approved its final version, and agreed to be accountable for the content of the study.

## Conflict of Interest

IF has received funding from the European Commission (grant agreement numbers 611223, 668259, and 825572) and speaking/congress fees from Dräger AG & Co KGaA outside the submitted work. ZZ has received a consulting fee from Dräger AG & Co KGaA outside the submitted work. The remaining authors declare that the research was conducted in the absence of any commercial or financial relationships that could be construed as a potential conflict of interest.

## Publisher’s Note

All claims expressed in this article are solely those of the authors and do not necessarily represent those of their affiliated organizations, or those of the publisher, the editors and the reviewers. Any product that may be evaluated in this article, or claim that may be made by its manufacturer, is not guaranteed or endorsed by the publisher.
