# Supplementary material for: Spatial Ventilation Inhomogeneity Determined by Electrical Impedance Tomography in Patients With Chronic Obstructive Lung Disease
Source: Front Physiol. 2021 Dec 13;12:762791. doi: 10.3389/fphys.2021.762791 (PMC8712108; doi:10.3389/fphys.2021.762791)
Supplement: Supplementary file 1 [file Table_1.pdf]

Supplemental Table 1 Results of conventional spirometry in the studied patients with COPD grouped according to the documented GOLD disease severity stage

Stage	**FEV_1/FVC (Z score)**	**FEV_1 (Z score)**	**FVC (Z score)**
GOLD I (n = 1)	-0.28	-1.42	-1.32
GOLD II (n = 13)	-1.91 ± 1.08	-2.59 ± 0.30	-1.73 ± 0.91
GOLD III (n = 21)	-3.24 ± 0.68	-3.47 ± 0.65	-2.36 ± 0.86
GOLD IV (n = 27)	-4.02 ± 0.68	-4.61 ± 0.60	-3.87 ± 0.82

Data are presented as single values or mean ± SD.

GOLD, Global Initiative for Chronic Obstructive Lung Disease; FEV_1, forced expiratory volume in 1s; FVC, forced vital capacity.
